# Sarcopenia and Mortality Risk of Patients with Sepsis: A Meta-Analysis

**DOI:** 10.1155/2022/4974410

**Published:** 2022-01-31

**Authors:** Wei Liu, Chenghuan Hu, Shuangping Zhao

**Affiliations:** ^1^Department of Critical Care Medicine, Xiangya Hospital of Central South University, Changsha, Hunan 410008, China; ^2^Hunan Provincial Clinical Research Center for Critical Care Medicine, Xiangya Hospital of Central South University, Changsha, Hunan 410008, China; ^3^National Clinical Research Center for Geriatric Disorders, Xiangya Hospital of Central South University, Changsha, Hunan 410008, China

## Abstract

**Background:**

The association between sarcopenia at admission and mortality in patients with sepsis has not been comprehensively evaluated. We performed a meta-analysis to systematically evaluate the above association.

**Methods:**

This meta-analysis included relevant observational studies from Medline, Embase, and Web of Science databases. A random-effect model after incorporation of the intrastudy heterogeneity was selected to pool the results. Subgroup analyses were applied to evaluate the influences of study characteristics on relationship.

**Results:**

Ten cohort studies including 2396 patients with sepsis were included, and 1496 (62.4%) of them had sarcopenia at presentation. Pooled results showed that compared to those without sarcopenia, septic patients with sarcopenia had a significantly increased early (in-hospital or 1-month) mortality risk (risk ration (RR): 2.14, 95% confidence interval (CI): 1.60–2.87, *P* < 0.001; *I*^2^ = 46%). Subgroup analyses showed consistent association between sarcopenia and increased acute mortality risk in septic patients which were not affected by study characteristics such as study design, country of the study, clinical settings, diagnostic criteria for sepsis, age, gender of the patients, and methods for diagnosis of sarcopenia (*P* for all subgroup analyses >0.05). Further meta-analyses showed that sarcopenia was also associated with increased mortality risk in septic patients at 3–6 months (RR: 2.13, 95% CI: 1.58–2.89, *P* < 0.001; *I*^2^ = 0%) and at 1 year (RR: 1.57, 95% CI: 1.09–2.24, *P* = 0.01; *I*^2^ = 29%).

**Conclusions:**

Current evidence suggests that sarcopenia may be a predictor of mortality in patients with sepsis.

## 1. Introduction

Sepsis is a common comorbidity in patients with critical illness, which has become a key determinant of the prognosis in these patients [1, 2]. Despite continuous efforts in the prevention of sepsis in critically ill patients, the incidence of sepsis in real-world acute clinical settings remains high, possibly due to multiple factors such as the accelerated aging of the global population, increased use of immunosuppressants, emerged antibiotic resistance, and frequently used invasive monitoring and treatment strategies [3, 4]. Since the pathogenesis of sepsis is complicated and the effective treatments for sepsis are limited, the mortality of patients with sepsis is very high, varying between 30% and 90% according to previous studies [5, 6]. Therefore, identification of risk factors for the mortality of patients with sepsis is important not only for the early risk stratification of the patients but also for the development of possible treatment strategies [7].

Sarcopenia, defined as an age-related generalized loss of skeletal muscle mass, has been related to frailty, overall functional impairment, and poor survival in geriatric population [8–10]. Although the universal measurements for sarcopenia remain to be established [11], sarcopenia has been proposed as a predictor of poor prognosis in patients of various chronic diseases, such as cancer [12], coronary artery disease [13], and chronic kidney disease [14]. Interestingly, patients with sepsis are also vulnerable to muscle catabolism, muscle weakness, and progressive muscle loss with the progression of the disease [15]. However, previous studies evaluating the association between sarcopenia at admission and mortality risk in patients with sepsis showed inconsistent results [16–25]. Particularly, the possible association between sarcopenia and early mortality has not been comprehensively evaluated [26]. Therefore, we performed a meta-analysis to systematically evaluate the association between sarcopenia at admission and mortality risk in patients with sepsis.

## 2. Methods

We followed the Meta-analysis of Observational Studies in Epidemiology (MOOSE) [27] and Cochrane's Handbook [28] guidelines during the design, performing, and presenting of the meta-analysis.

### 2.1. Search of Electronic Databases

We identified studies by a systematic search of Medline, Embase, and Web of Science electronic databases using the following terms: (1) “sarcopenia” OR “muscle wasting” OR “muscle loss” OR “muscular atrophy” OR “muscle depletion” OR “sarcopaenia” OR “sarcopenic” OR “presarcopenia” OR “sarcopaenic” OR “lean body mass” OR “cross-sectional muscle area” OR “skeletal muscle depletion” OR “muscle mass” OR “muscle index” and (2) “sepsis” OR “septicemia” OR “septic.” Only clinical studies published in English were selected. An additional manual check-up for the reference lists of relevant original and review articles was performed as supplement. The last literature search was conducted on July 28, 2021.

### 2.2. Selection of Eligible Studies

Inclusion criteria were (1) observational studies published as full-length articles; (2) included adult patients (18 years or above) with confirmed diagnosis of sepsis; (3) sarcopenia identified at presentation and considered as exposure; (4) incidence of mortality was reported as outcome of interest; and (5) reported the association between sarcopenia and the risk of mortality during the follow-up duration. The diagnostic method and criteria of sarcopenia was consistent with the criteria adopted in the original articles. Reviews, preclinical studies, studies that did not include patients with sepsis, or studies that did not report mortality during follow-up durations were excluded.

### 2.3. Extraction of Data and Evaluation of Study Quality

Two of the authors independently conducted electronic database search, extraction of study data, and assessment of study quality according to the inclusion criteria described above. If there were discrepancies, they were resolved by consensus between the authors. The extracted data included the following: (1) name of the first author, year of the publication, study design, country, and clinical settings of the study; (2) population characteristics, including the diagnostic criteria for sepsis, total number, mean age, and sex of the patients; (3) methods and cutoff values for the diagnosis of sarcopenia, and number of patients with sarcopenia at baseline; and (4) follow-up durations and variables adjusted in the multivariate model analyzing the association between sarcopenia and mortality risk of patients with sepsis. The Newcastle–Ottawa Scale [29] was used for study quality assessment, which included three domains such as defining of study groups, between-group comparability, and validation of the outcome. This scale totally scored from 1 to 9 stars, with 9 stars indicating the highest study quality level.

### 2.4. Statistical Methods

The primary objective of the study was to determine the association between sarcopenia and early mortality (in-hospital or 1-month mortality) risk in patients with sepsis. The secondary objective was to determine the relationship of sarcopenia and 3–6 months and 1 year mortality of septic patients. Risk ratios (RRs) and 95% confidence intervals (CIs) were selected as the general outcome variable for the relationship between sarcopenia and risk of mortality in patients with sepsis compared to those without sarcopenia at presentation. Data of RRs and standard errors (SEs) were calculated from 95% CIs or *P* values, and an additional logarithmical transformation was performed to stabilize variance and normalize to the distribution [28]. The Cochrane's *Q* test was used to evaluate the heterogeneity, and the *I*^2^ statistic was also estimated [30]. Heterogeneity was deemed to be significant if *I*^2^ > 50%. We used a random-effect model for data synthesis because this model has incorporated the potential between-study heterogeneity and could provide a more generalized result [28]. Sensitivity analyses were performed by omitting one individual study at a time to examine the robustness of the finding [31]. Influences of study characteristics on the association between sarcopenia and early mortality were tested with predefined subgroup analyses. The funnel plots were constructed, and a visual inspection of the symmetry was conducted to reflect the publication bias. Egger's regression asymmetry test was further performed for the evaluation of potential publication bias [32]. We used the RevMan (Version 5.1; Cochrane Collaboration, Oxford, UK) software for the statistical analyses.

## 3. Results

### 3.1. Results of Database Search

**Figure 1:**
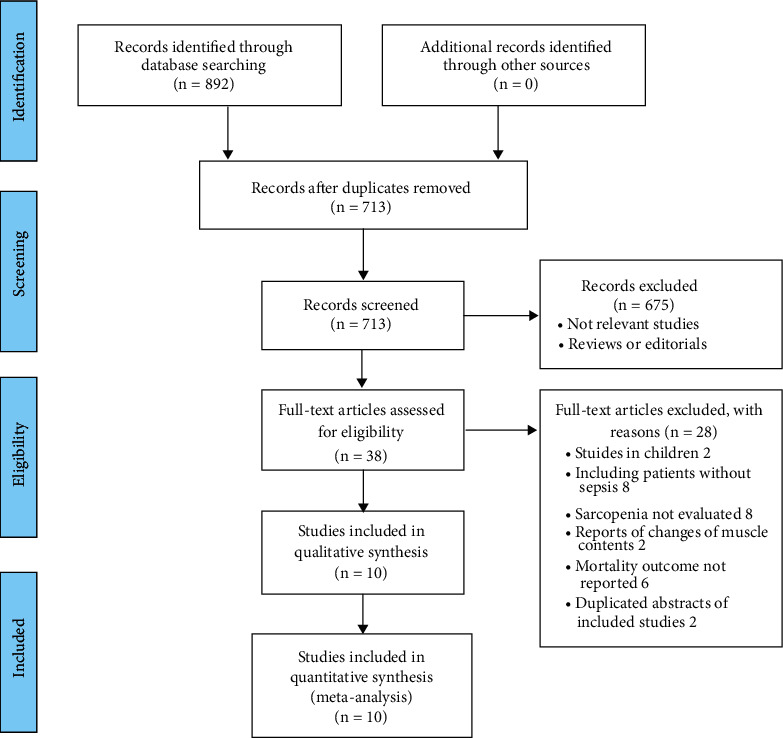
Flowchart of the database search and study identification.

The database search process is summarized in Figure 1. Briefly, 892 articles were found in the initial literature search of the Medline, Embase, and Web of Science databases; after excluding the duplications, 713 studies remained. An additional 675 were excluded through screening of the titles and abstracts mainly because of the irrelevance to the meta-analysis. The remaining 38 studies underwent a full-text review. Of the 38 studies, 28 were further excluded for the reasons shown in Figure 1. Finally, 10 cohort studies [16–25] were included.

### 3.2. Characteristics of the Included Studies

As given in Table 1, 10 cohort studies [16–25] including 2396 adult patients with sepsis were included in the meta-analysis. These studies were published between 2017 and 2021 and performed in Japan [16, 18, 25], Korea [19, 21–23], China [17], Italy [20], and the United States [24]. All of the included studies were retrospective cohort studies, except for one study, which was a prospective cohort study [24]. The studies included patients with sepsis admitted in emergency department or intensive care unit diagnosed with the sepsis-1 [20], sepsis-2 [17], and sepsis-3 criteria [16, 18, 19, 21–25], respectively. In most of the included studies, sarcopenia was diagnosed with skeletal muscle index (SMI) [17, 19, 21–25] or skeletal muscle area (SMA) [16, 18] with computed tomography (CT), while in one study, sarcopenia was diagnosed with measuring of midarm muscle circumference [20]. Overall, 1496 (62.4%) of the included patients had sarcopenia at presentation. The follow-up durations varied from within hospitalization to one year after discharge. Variables such as age, sex, comorbidities, biomarkers for systemic inflammation, and severity scores for sepsis were adjusted to a varying degree among the included studies when the association between sarcopenia and mortality risk in septic patients was analyzed. The NOS of the included studies were 7–9 stars, suggesting good quality of all included studies (Table 2).

### 3.3. Meta-Analysis Results

**Figure 2:**
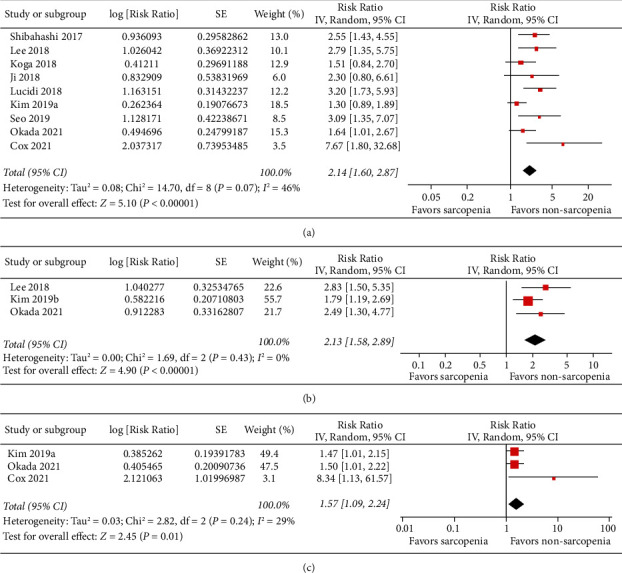
Forest plots for the meta-analysis of the association between sarcopenia and mortality risk in patients with sepsis. (a) Association between sarcopenia and early mortality risk. (b) Association between sarcopenia and mortality risk at 3–6 months after discharge. (c) Association between sarcopenia and mortality risk at 1 year after discharge.

Moderate heterogeneity was observed among the nine studies [16–21, 23–25] that reported the association between sarcopenia and early mortality risk in patients with sepsis (*P* for Cochrane's *Q* test = 0.07, *I*^2^ = 46%). Pooled results with a random-effect model showed that septic patients with sarcopenia had a significantly increased risk of acute mortality as compared to those without sarcopenia (RR: 2.14, 95% CI: 1.60–2.87, *P* < 0.001; Figure 2(a)). Sensitivity analyses by excluding one study at a time showed consistent results (RR: 2.01–2.33, *P* for all subgroup analyses <0.05). Subgroup analyses showed consistent association between sarcopenia and increased early mortality risk in septic patients which were not affected by study characteristics such as study design, country of the study, clinical settings, diagnostic criteria for sepsis, age, gender of the patients, methods for diagnosis of sarcopenia, in in-hospital and 1-month mortality, and in different quality scores (*P* for all subgroup analyses >0.05; Table 3). Further meta-analyses showed that sarcopenia was also associated with increased mortality risk in septic patients at 3–6 months (3 studies [19, 22, 25], RR: 2.13, 95% CI: 1.58–2.89, *P* < 0.001; *I*^2^ = 0%; Figure 2(b)) and at 1 year (three studies [21, 24, 25], RR: 1.57, 95% CI: 1.09–2.24, *P* = 0.01; *I*^2^ = 29%; Figure 2(c)) after discharge.

### 3.4. Publication Bias

**Figure 3:**
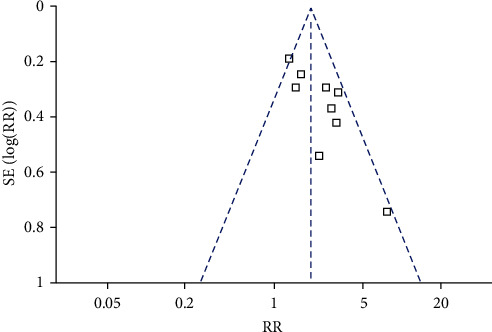
Funnel plots for the publication bias underlying the meta-analysis of the association between sarcopenia and early mortality risk.

Figure 3 shows the funnel plots regarding the relationship between sarcopenia and mortality risk in patients with sepsis. Visual inspection found symmetry of the plots, which suggested a low risk of publication bias. Results of Egger's regression tests also suggested low risk of publication bias (*P* = 0.377). The publication biases for the meta-analyses of the mortality at 3–6 months and 1 year after discharge were difficult to estimate because only three studies were available.

## 4. Discussion

In this meta-analysis, by pooling the results of available cohort studies, we found that sarcopenia at admission was associated with an increased early mortality risk in patients with sepsis. Besides, further sensitivity and subgroup analyses showed consistent association between sarcopenia and higher early mortality in septic patients, which were not driven by either of the included studies, or significantly affected by study and patient characteristics, such as study design, country of the study, clinical settings, diagnostic criteria for sepsis, age, gender of the patients, methods for diagnosis of sarcopenia, in in-hospital and 1-month mortality, or different quality scores. In addition, meta-analyses with limited datasets also suggested that sarcopenia was associated with a higher mortality of septic patients at 3–6 months and 1 year after discharge. Taken together, current evidence from epidemiological studies suggested that sarcopenia at admission may be a predictor of increased mortality risk in patients with sepsis. Although these findings should be validated in large-scale prospective cohort studies, evaluation for sarcopenia at admission may be important for the risk stratification of patients with sepsis.

To the best of our knowledge, this study may be the first meta-analysis that evaluated the association between sarcopenia and mortality risk in patients with sepsis. Our study has a few strengths in methodology. Firstly, all of the included studies were cohort studies, which therefore enabled us to provide a temporal relationship between sarcopenia and increased mortality risk in patients with sepsis. Secondly, multivariate analyses were applied among the included studies when the associations between sarcopenia and mortality risk in septic patients were estimated. The results obtained after the adjustment of the possible related factors may indicate a possible independent association between sarcopenia and higher mortality in septic patients. This is important because sarcopenia and sepsis may share some common risk factors, such as aging [33, 34]. In addition, we analyzed the association between sarcopenia and early and late mortality in septic patients separately because sepsis is associated with a relatively higher early mortality, and the risk factors for early and late mortality in septic patients may be different [35, 36]. However, the results showed that sarcopenia was consistently associated with a higher early mortality (in-hospital/1 month) and a late mortality (3–6 months and 1 year) in patients with sepsis. Finally, we performed multiple sensitivity and subgroup analyses to validate the findings, which were not significantly influenced by either of the included study or the characteristics of study and patients. Taken together, results of this meta-analysis suggested that sarcopenia at admission may be a predictor of increased mortality risk in patients with sepsis. The mechanisms underlying the association between sarcopenia and higher mortality risk in patients with sepsis remain to be determined. In general, sarcopenia has been considered as an indicator of physical aging, senescence of the immune system, and poor host's response to infection [37, 38], and the latter plays a key role in the pathogenesis and deterioration of sepsis. Besides, sarcopenia may also reflect the poor nutritional status and the overall frailty of the patients [39], which also adversely influenced the prognosis in patients with sepsis [40, 41]. Besides, sarcopenia has been associated with poor immune response, metabolic stress, and impaired respiratory and swallowing function during an acute clinical setting such as severe infection [42], which all lead to the increased mortality risk in these patients. The exact molecular mechanisms underlying the association between sarcopenia and mortality in sepsis should be further investigated.

Our study has limitations which should be considered when the results of the meta-analysis are interpreted. Firstly, most of the included studies were retrospective, which exposed the findings of the meta-analysis to the risks of recall and selection biases. Therefore, large-scale prospective studies are needed to validate the findings. Secondly, we could not exclude the possibility that some other clinical factors may confound the possible association between sarcopenia and mortality in septic patients, such as the nutritional and frailty status of the patients, which were rarely adjusted among the included studies. In addition, the optimal measuring methods and cutoffs for the defining of sarcopenia in an acute clinical setting such as in patients with sepsis remains to be determined. Most of the included studies used the cross-sectional view of the muscle obtained by CT to calculate the skeletal muscle mass of the patients because CT is frequently required in patients with sepsis as a part of the initial examination. However, the cutoffs for defining of sarcopenia based on this method vary among the included studies, which may contribute to the heterogeneity. A standard and universal definition of sarcopenia is needed in this regard [43]. Finally, a causative relationship between sarcopenia and higher mortality risk in septic patients could not be determined from our study because this is a meta-analysis based on observational studies. Clinical trials may be considered to evaluate the influences of intervention targeting sarcopenia on clinical outcomes in patients with sepsis.

## 5. Conclusions

In conclusion, results of this meta-analysis showed that sarcopenia at admission may be a predictor of increased mortality risk in patients with sepsis. Although these findings should be validated in large-scale prospective cohort studies, evaluation for sarcopenia at admission may be important for the risk stratification of patients with sepsis.

## Figures and Tables

**Table 1:** Characteristics of the included studies.

Study	Design	Country	Clinical setting	Diagnostic criteria for sepsis	Sample size	Mean age (years)	Male (%)	Diagnosis of sarcopenia	Cutoff	Patients with sarcopenia at baseline	Follow-up duration	Variables adjusted	NOS
Shibahashi [16]	RC	Japan	ICU	Sepsis-3	150	75	69	SMA (CT)	<45.2 cm^2^ for male and <39.0 cm^2^ for female	83	Within hospitalization	Age, sex, and APACHE II score	8
Lee [19]	RC	Korea	ED	Sepsis-3	274	70.9	46	SMI (CT)	<545 mm^2^/m^2^ for male and <385 mm^2^/m^2^ for female	77	28 days and 6 months	Age, sex, comorbidities, CRP, PCT, site of infection, and SOFA	8
Koga [18]	RC	Japan	ICU	Sepsis-3	191	71.8	66	SMA (CT)	<80% of predicted value	91	Within hospitalization	Age and sex	7
Ji [17]	RC	China	ICU	Sepsis-2	236	67.8	58.9	SMI (CT)	<40.8 cm^2^/m^2^ for male and <34.9 cm^2^/m^2^ for female	114	30 days	Age, sex, use of vasopressor, mixed organism, and APACHE II score or SOFA score	8
Lucidi [20]	RC	Italy	ICU	Sepsis-1	74	49.7	75.7	MAMC	<95% of predicted value	32	Within hospitalization	Age, sex, comorbidities, BUN, SCr, and serum albumin	7
Kim [9]	RC	Korea	ICU	Sepsis-3	516	68.6	67.5	SMI (CT)	<55 cm^2^/m^2^ for male and <39 cm^2^/m^2^ for female	421	Within hospitalization and 12 months	Age, sex, RRT, use of vasopressor, CCI, and SOFA score	9
Kim [19]	RC	Korea	ED	Sepsis-3	478	65	62.1	SMI (CT)	<43 cm^2^/m^2^ for a BMI <25 kg/m^2^, <53 cm^2^/m^2^ for a BMI of 25 kg/m^2^ or more for male, and <41 cm^2^/m^2^ regardless of the BMI for female	419	3 months	Age, sex, comorbidities, site of infection, and SOFA score	8
Seo [23]	RC	Korea	ED	Sepsis-3	175	65	62.9	SMI (CT)	<43 cm^2^/m^2^ for a BMI <25 kg/m^2^, <53 cm^2^/m^2^ for a BMI of 25 kg/m^2^ or more for male, and <41 cm^2^/m^2^ regardless of the BMI for female	151	28 days	Age, sex, comorbidities, serum lactate, and SOFA score	7
Okada [25]	RC	Japan	ICU	Sepsis-3	255	76	66.3	SMI (CT)	T3 : T1 of psoas index	85	Within hospitalization, 3 months, and 12 months	Age, sex, infection site, CCI, and SOFA score	9
Cox [24]	PC	USA	ICU	Sepsis-3	47	53.1	29.8	SMI (CT)	<52.4 cm^2^/m^2^ for male and <38.5 cm^2^/m^2^ for female	23	Within hospitalization and 12 months	Age, sex, CCI, and severity of sepsis	8

PC, prospective cohort; RC, retrospective cohort; NOS, Newcastle–Ottawa Scale; ED, emergency department; ICU, intensive care unit; SMI, skeletal muscle index; SMA, skeletal muscle area; MAMC, midarm muscle circumference; CT, computed tomography; BMI, body mass index; APACHE II, Acute Physiology and Chronic Health Evaluation 2; CRP, C-reactive protein; PCT, procalcitonin; *T*, tertile; SOFA, Sequential Organ Failure Assessment; CCI, Charlson Comorbidity Index; RRT, renal replacement therapy; BUN, blood urea nitrogen; SCr, serum creatinine.

**Table 2:** Details of quality evaluation of the included studies via the Newcastle–Ottawa Scale.

Study	Representativeness of the exposed cohort	Selection of the nonexposed cohort	Ascertainment of exposure	Outcome not present at baseline	Control for age and sex	Control for other confounding factors	Assessment of outcome	Enough long follow-up duration	Adequacy of follow-up of cohorts	Total
Shibahashi [16]	1	1	1	1	1	1	1	0	1	8
Lee [19]	1	1	1	1	1	1	1	1	0	8
Koga [18]	1	1	1	1	1	0	1	0	1	7
Ji [17]	1	1	1	1	1	1	1	0	1	8
Lucidi [20]	0	1	1	1	1	1	1	0	1	7
Kim [9]	1	1	1	1	1	1	1	1	1	9
Kim [19]	0	1	1	1	1	1	1	1	1	8
Seo [23]	0	1	1	1	1	1	1	0	1	7
Okada [25]	1	1	1	1	1	1	1	1	1	9
Cox [24]	0	1	1	1	1	1	1	1	1	8

**Table 3:** Results of subgroup analyses for the association between sarcopenia and acute mortality of patients with sepsis.

Study characteristics	Datasets number	RR (95% CI)	*I* ^2^	*P* for the subgroup effect	*P* for subgroup difference
Design					
Prospective	1	2.14 (1.60, 2.87)	NA	0.006	
Retrospective	8	7.67 (1.80, 32.68)	37%	<0.001	0.08
Country					
Asian	7	1.85 (1.43, 2.40)	25%	<0.001	
Non-Asian	2	3.83 (1.92, 7.67)	16%	<0.001	0.07
Clinical settings					
ED	2	2.92 (1.69, 5.03)	0%	<0.001	
ICU	7	2.01 (1.44, 2.81)	51%	<0.001	0.25
Diagnosis of sepsis					
Sepsis-1 or sepsis-2	2	2.94 (1.73, 5.01)	0%	<0.001	
Sepsis-3	7	2.01 (1.45, 2.79)	49%	<0.001	0.23
Mean age					
<70 years	5	2.54 (1.43, 4.53)	66%	0.002	
70 years or above	4	1.95 (1.46, 2.60)	0%	<0.001	0.42
Proportion of men					
<65%	4	3.07 (1.94, 4.86)	0%	<0.001	
65% or above	5	1.84 (1.32, 2.55)	50%	<0.001	0.07
Diagnosis of sarcopenia					
SMI	6	2.11 (1.41, 3.16)	51%	<0.001	
SMA	2	1.96 (1.17, 3.28)	36%	0.01	
MAMC	1	3.20 (1.73, 5.93)	NA	<0.001	0.44
Duration					
In-hospital mortality	6	2.01 (1.39, 2.90)	58%	<0.001	
1-month mortality	3	2.77 (1.71, 4.50)	0%	<0.001	0.30
Quality score					
NOS = 7	3	2.38 (1.42, 3.98)	44%	= 0.001	
NOS = 8	4	2.81 (1.88, 4.19)	0%	<0.001	
NOS = 9	2	1.42 (1.05, 1.91)	0%	0.02	0.08

RR, risk ratio; CI, confidence interval; NA, not applicable; ED, emergency department; ICU, intensive care unit; SMI, skeletal muscle index; SMA, skeletal muscle area; MAMC, midarm muscle circumference; NOS, Newcastle–Ottawa Scale.

## Data Availability

The data used to support the findings of this study are available from the corresponding author upon request.

## Additional Points

A comprehensive search strategy using combined keywords was designed. Electronic databases that included PubMed, Embase, and Web of Science were searched up to July 28, 2021. Observational studies reporting the association sarcopenia and mortality in patients with sepsis were included for review. *Message for the Clinic*. Sarcopenia is associated with a higher risk of early mortality in septic patients. The association between sarcopenia and mortality in septic patients remains at 3–6 months and 1 year after discharge. Evaluation for sarcopenia at admission may be important for the risk stratification of patients with sepsis.

## References

[1] Chiu C., Legrand M. (2021). Epidemiology of sepsis and septic shock. *Current Opinion in Anaesthesiology*.

[2] Evans L., Rhodes A., Alhazzani W. (2021). Surviving sepsis campaign: international guidelines for management of sepsis and septic shock 2021. *Intensive Care Medicine*.

[3] Font M. D., Thyagarajan B., Khanna A. K. (2020). Sepsis and Septic Shock - basics of diagnosis, pathophysiology and clinical decision making. *Medical Clinics of North America*.

[4] Markwart R., Saito H., Harder T. (2020). Epidemiology and burden of sepsis acquired in hospitals and intensive care units: a systematic review and meta-analysis. *Intensive Care Medicine*.

[5] Fleischmann-Struzek C., Mellhammar L., Rose N. (2020). Incidence and mortality of hospital- and ICU-treated sepsis: results from an updated and expanded systematic review and meta-analysis. *Intensive Care Medicine*.

[6] Shappell C. N., Klompas M., Rhee C. (2020). Surveillance strategies for tracking sepsis incidence and outcomes. *The Journal of Infectious Diseases*.

[7] van der Slikke E. C., An A. Y., Hancock R. E. W., Bouma H. R. (2020). Exploring the pathophysiology of post-sepsis syndrome to identify therapeutic opportunities. *EBioMedicine*.

[8] Roberts S., Collins P., Rattray M. (2021). Identifying and managing malnutrition, frailty and sarcopenia in the community: a narrative review. *Nutrients*.

[9] Kim J. W., Kim R., Choi H., Lee S. J., Bae G. U. (2021). Understanding of sarcopenia: from definition to therapeutic strategies. *Archives of Pharmacal Research*.

[10] Cannataro R., Carbone L., Petro J. L. (2021). Sarcopenia: etiology, nutritional approaches, and miRNAs. *International Journal of Molecular Sciences*.

[11] Dent E., Woo J., Scott D., Hoogendijk E. O. (2021). Sarcopenia measurement in research and clinical practice. *European Journal of Internal Medicine*.

[12] Xia L., Zhao R., Wan Q. (2020). Sarcopenia and adverse health-related outcomes: an umbrella review of meta-analyses of observational studies. *Cancer Med*.

[13] Xue Q., Wu J., Ren Y., Hu J., Yang K., Cao J. (2021). Sarcopenia predicts adverse outcomes in an elderly population with coronary artery disease: a systematic review and meta-analysis. *BMC Geriatrics*.

[14] Santana Gomes T., Espirito Santo Silva D. D., Xavier Junior G. F., de Farias Costa P. R., Gusmao Sena M. H. L., Barreto Medeiros J. M. (2021). Sarcopenia and mortality in patients with chronic non-dialytic renal disease: systematic review and meta-analysis. *Journal of Renal Nutrition*.

[15] Mankowski R. T., Laitano O., Clanton T. L., Brakenridge S. C. (2021). Pathophysiology and treatment strategies of acute myopathy and muscle wasting after sepsis. *Journal of Clinical Medicine*.

[16] Shibahashi K., Sugiyama K., Kashiura M., Hamabe Y. (2017). Decreasing skeletal muscle as a risk factor for mortality in elderly patients with sepsis: a retrospective cohort study. *J Intensive Care*.

[17] Ji Y., Cheng B., Xu Z. (2018). Impact of sarcopenic obesity on 30-day mortality in critically ill patients with intra-abdominal sepsis. *Journal of Critical Care*.

[18] Koga Y., Fujita M., Yagi T. (2018). Early enteral nutrition is associated with reduced in-hospital mortality from sepsis in patients with sarcopenia. *Journal of Critical Care*.

[19] Lee Y., Park H. K., Kim W. Y., Kim M. C., Jung W., Ko B. S. (2018). Muscle mass depletion associated with poor outcome of sepsis in the emergency department. *Annals of Nutrition & Metabolism*.

[20] Lucidi C., Lattanzi B., Di Gregorio V. (2018). A low muscle mass increases mortality in compensated cirrhotic patients with sepsis. *Liver International*.

[21] Kim T., Huh S., Kim S. Y. (2019). ICU rehabilitation is associated with reduced long-term mortality from sepsis in patients with low skeletal muscle mass: a case control study. *Annals of Translational Medicine*.

[22] Kim Y. J., Seo D. W., Kang J., Huh J. W., Kim K. W., Kim W. Y. (2019). Impact of body composition status on 90-day mortality in cancer patients with septic shock: sex differences in the skeletal muscle index. *Journal of Clinical Medicine*.

[23] Seo D. W., Kim K. W., Sohn C. H. (2019). Progressive loss of muscle mass could be an adverse prognostic factor of 28-day mortality in septic shock patients. *Scientific Reports*.

[24] Cox M. C., Booth M., Ghita G. (2021). The impact of sarcopenia and acute muscle mass loss on long-term outcomes in critically ill patients with intra-abdominal sepsis. *Journal of Cachexia Sarcopenia Muscle*.

[25] Okada Y., Kiguchi T., Okada A., Iizuka R., Iwami T., Ohtsuru S. (2021). Predictive value of sarcopenic findings in the psoas muscle on CT imaging among patients with sepsis. *The American Journal of Emergency Medicine*.

[26] Schefold J. C., Wollersheim T., Grunow J. J., Luedi M. M., Z’Graggen W. J., Weber-Carstens S. (2020). Muscular weakness and muscle wasting in the critically ill. *J Cachexia Sarcopenia Muscle*.

[27] Stroup D. F., Berlin J. A., Morton S. C. (2000). Meta-analysis of observational studies in epidemiology: a proposal for reporting. Meta-analysis of Observational Studies in Epidemiology (MOOSE) group. *The Journal of the American Medical Association*.

[28] Higgins J., Green S. (2011). Cochrane handbook for systematic reviews of interventions version 5.1.0. *The Cochrane Collaboration*.

[29] Wells G. A., Shea B., O’Connell D. (2010). The newcastle-ottawa scale (nos) for assessing the quality of nonrandomised studies in meta-analyses. http://www.ohri.ca/programs/clinical_epidemiology/oxford.asp.

[30] Higgins J. P., Thompson S. G. (2002). Quantifying heterogeneity in a meta-analysis. *Statistics in Medicine*.

[31] Patsopoulos N. A., Evangelou E., Ioannidis J. P. (2008). Sensitivity of between-study heterogeneity in meta-analysis: proposed metrics and empirical evaluation. *International Journal of Epidemiology*.

[32] Egger M., Davey Smith G., Schneider M., Minder C. (1997). Bias in meta-analysis detected by a simple, graphical test. *British Medical Association*.

[33] Larsson L., Degens H., Li M. (2019). Sarcopenia: aging-related loss of muscle mass and function. *Physiological Reviews*.

[34] Rowe T. A., McKoy J. M. (2017). Sepsis in older adults. *Infectious Disease Clinics of North America*.

[35] Gotts J. E., Matthay M. A. (2016). Sepsis: pathophysiology and clinical management. *British Medical Association*.

[36] Shankar-Hari M., Ambler M., Mahalingasivam V., Jones A., Rowan K., Rubenfeld G. D. (2016). Evidence for a causal link between sepsis and long-term mortality: a systematic review of epidemiologic studies. *Critical Care*.

[37] Nelke C., Dziewas R., Minnerup J., Meuth S. G., Ruck T. (2019). Skeletal muscle as potential central link between sarcopenia and immune senescence. *EBioMedicine*.

[38] Wilson D., Jackson T., Sapey E., Lord J. M. (2017). Frailty and sarcopenia: the potential role of an aged immune system. *Ageing Research Reviews*.

[39] Cruz-Jentoft A. J., Kiesswetter E., Drey M., Sieber C. C. (2017). Nutrition, frailty, and sarcopenia. *Aging Clinical and Experimental Research*.

[40] De Waele E., Malbrain M., Spapen H. (2020). Nutrition in sepsis: a bench-to-bedside review. *Nutrients*.

[41] Martin S., Perez A., Aldecoa C. (2017). Sepsis and immunosenescence in the elderly patient: a review. *Frontiers of Medicine*.

[42] Wang P. Y., Li Y., Wang Q. (2021). Sarcopenia: an underlying treatment target during the COVID-19 pandemic. *Nutrition*.

[43] Cruz-Jentoft A. J., Bahat G., Bauer J. (2019). Sarcopenia: revised European consensus on definition and diagnosis. *Age and Ageing*.

